# Estratégia Fármaco-Invasiva no Infarto do Miocárdio: Análise Descritiva, Apresentação de Sintomas Isquêmicos e Preditores de Mortalidade

**DOI:** 10.36660/abc.20211055

**Published:** 2022-11-01

**Authors:** Henrique Tria Bianco, Rui Povoa, Maria Cristina Izar, Claudia Maria Rodrigues Alves, Adriano Henrique Pereira Barbosa, Maria Teresa Nogueira Bombig, Iran Gonçalves, Bráulio Luna, Ana Caroline Aguirre, Pedro Ivo de Marqui Moraes, Dirceu Almeida, Flávio Tocci Moreira, Fernando Focaccia Povoa, Edson Stefanini, Adriano Mendes Caixeta, Amanda S. Bacchin, Valdir Ambrósio Moisés, Francisco A.H. Fonseca

**Affiliations:** 1 Universidade Federal de São Paulo Cardiologia São Paulo SP Brasil Universidade Federal de São Paulo – Cardiologia, São Paulo, SP – Brasil; 2 Universidade Federal de São Paulo Escola Paulista de Medicina Medicina São Paulo SP Brasil Universidade Federal de São Paulo Escola Paulista de Medicina – Medicina, São Paulo, SP – Brasil

**Keywords:** Infarto do Miocárdio por Supradesnivelamento do Segmento ST, Síndrome Coronáriana Aguda, Intervenção Coronária Percutânea/métodos, Terapia Trombolítica/métodos Angina Pectoris, Hospitalização, Mortalidade

## Abstract

**Fundamento:**

O infarto do miocárdio com elevação do segmento-ST (IAMCSST) é definido por sintomas acompanhados por alterações típicas do eletrocardiograma. Entretanto, a caracterização dos sintomas isquêmicos não é clara, principalmente em subgrupos, como mulheres e idosos.

**Objetivos:**

Analisar a tipificação dos sintomas isquêmicos, métricas temporais e observar a ocorrência de desfechos intra-hospitalares, em análise dos escores preditivos, em pacientes com IAMCSST, em estratégia fármaco-invasiva.

**Métodos:**

Estudo envolvendo 2.290 pacientes. Tipos de apresentações clínicas pré-definidas: dor típica, dor atípica, dispnéia, sincope. Medimos o tempo entre o início dos sintomas à demanda pelo atendimento e o intervalo entre a chegada à unidade-médica e trombólise. *Odds-ratios* (OR; IC-95%) foram estimadas em modelo de regressão. Curvas ROCs foram construídas para preditores de mortalidade. Nível de significância adotado (alfa) foi de 5%.

**Resultados:**

Mulheres apresentaram alta prevalência de sintomas atípicos; maior tempo entre o início dos sintomas e a procura por atendimento; atraso entre a chegada ao pronto-socorro e a fibrinólise. A mortalidade hospitalar foi de 5,6%. Predição de risco pela classificação Killip-Kimball: AUC: [0,77 (0,73-0,81)] em classe ≥II. Subgrupos estudados [OR (IC-95%)]: mulheres [2,06 (1,42-2,99); p=0,01]; insuficiência renal crônica [3,39 (2,13-5,42); p<0,001]; idosos [2,09 (1,37-3,19) p<0,001]; diabéticos [1,55 (1,04-2,29); p=0,02]; obesos 1,56 [(1,01-2,40); p=0,04]; acidente vascular cerebral prévio [2,01 (1,02-3,96); p=0,04] correlacionaram-se com maiores taxas de mortalidade.

**Conclusão:**

Apesar das mais altas taxas de mortalidade em alguns subgrupos, disparidade significativa persiste nas mulheres, com atrasos no reconhecimento dos sintomas e trombólise imediata. Destaca-se a aplicabilidade do escore Killip-Kimball na predição, independentemente da apresentação clínica.

## Introdução

Apesar dos avanços nas abordagens de reperfusão, o infarto agudo do miocárdio continua a ser a principal causa de morte em todo o mundo. Seu diagnóstico é considerado quando se pode detectar alterações típicas do eletrocardiograma (ECG) e/ou a elevação de marcadores, especialmente as troponinas, que recebem cada vez mais atenção como marcadores específicos de injúria miocárdica. O infarto do miocárdio com supradesnivelamento do segmento ST (IAMCSST) geralmente é causado por oclusão coronária aguda, secundária à ruptura de placa e trombose, e requer a intervenção precoce.^1^ Portanto, o controle do IAMCSST deve ser realizado o mais rápido possível para evitar maiores lesões ao miocárdio e diminuir o risco de complicações e mortalidade. Embora a intervenção coronária percutânea (ICP) seja considerada o “padrão-ouro” de tratamento, ela não está suficientemente disponível, especialmente nos países em desenvolvimento. O estudo STREAM valoriza uma estratégia de reperfusão combinando terapia fibrinolítica e transferência imediata a um centro terciário, para a realização da ICP de resgate em pacientes que não respondem à fibrinólise, bem como uma angiografia diagnóstica precoce e uma ICP secundária no período de 24 horas após a trombólise.^2^ Entretanto, alguns fatores podem influenciar a demora em procurar assistência emergencial, tais como a percepção e o reconhecimento de sintomas isquêmicos agudos. A interpretação de sinais de alerta é o gatilho que leva os pacientes a buscar a assistência médica, devido à gravidade dessa condição potencialmente fatal. Em contraste, a dor deve ser vista como um fenômeno multidimensional que envolve aspectos fisiológicos, sensoriais e socioculturais, e pode ser afetado por expectativas dentro de um contexto cultural.

A estratificação dos riscos permite aos prestadores identificar o nível certo de cuidado e serviços para subgrupos de pacientes distintos. Ela é o processo de atribuir um status de risco e, em seguida, usar esse relatório para orientar sobre que cuidado é oferecido para melhorar os desfechos gerais de saúde. No entanto, durante o processo diagnóstico de infarto do miocárdio, com base em relatórios clínicos e critérios de ECG, podem surgir diferenças em relação a como os sintomas são tratados, especialmente em subgrupos específicos, tais como grupos de mulheres ou de pessoas mais idosas, apresentando evidências e relevância de recanalização precoce, cujos benefícios se tornam mais discretos ou até inexistentes na reperfusão tardia.^3,4^ Além disso, ainda há controvérsias e interesses especiais em relação ao desempenho de preditores de mortalidade precoces em pacientes que passaram por tratamento trombolíticos em uma abordagem fármaco-invasiva. Portanto, o presente estudo considerou a avaliação estratificada de sintomas isquêmicos, fundamentalmente associados a métricas temporais, incluindo o tempo entre o aparecimento dos sintomas e a busca por tratamento médico, as necessidades médicas do paciente entre a chegada à unidade de emergência e o reconhecimento da condição aguda, com o início imediato dos protocolos de reperfusão. No cenário da estratégia fármaco-invasiva, pode-se também especular sobre possíveis diferenças na forma em que os sintomas apareceram e os tempos pivotais em alguns subgrupos, considerando o impacto da reperfusão tardia em desfechos clínicos relevantes.

Dessa forma, nosso estudo teve o objetivo de examinar as associações entre a apresentação de sintomas isquêmicos e fatores de risco com os desfechos cardiovasculares em uma coorte de pacientes com IAMCSST durante o período de hospitalização, bem como realizar uma análise precisa das classificações de previsão de risco.

## Métodos

### Desenho do estudo e declarações éticas

Estudo prospectivo e observacional, com um tamanho de amostra definido por conveniência, envolvendo 2.290 pacientes com IAMCSST que foram admitidos consecutivamente internados em um hospital universitário na cidade de São Paulo, Brasil. Todos os pacientes foram inicialmente submetidos a terapia trombolítica com tenecteplase (TNK) em unidades hospitalares e centros de cuidado primários e, em seguida, encaminhados à angiografia coronária. Quando apropriado, foram realizadas intervenções coronárias percutâneas no período de 24 horas após a fibrinólise, ou imediatamente, se houvesse a necessidade de tratamento de resgate. Este estudo está de acordo com a Declaração de Helsinki, o comitê de ética local aprovou o protocolo de pesquisa, e o consentimento informado foi obtido de todos os pacientes ou seus guardiões legais. O estudo está registrado em ClinicalTrials.gov (NCT02090712).

### Estratégia fármaco-invasiva

A estratégia fármaco-invasiva é definida pelo tratamento de fibrinólise com um bolus intravenoso de TNK, com uma dose ajustada pelo peso, seguida de cateterismo cardíaco no período de 24 horas, mesmo em pacientes estáveis com reperfusão bem-sucedida, com a intenção de tratar a lesão culpada. Após os resultados do estudo STREAM em junho de 2013, os pacientes com mais de 75 anos de idade receberam meia dose de tenecteplase (1/2 TNK). No diagnóstico, os pacientes receberam ácido acetilsalicílico e clopidogrel conforme recomendado pelas diretrizes.^5^ As angioplastias de resgate foram indicadas pela equipe médica local, devido a trombólise ineficaz no tratamento da artéria relacionada ao infarto (ARI). O termo *lesão culpada* foi usado para designar o vaso arterial responsável pelos sintomas do paciente com IAMCSST. Na maioria dos casos, apenas as lesões culpadas foram tratadas, ou seja, apenas lesões em ARI foram tratadas diretamente por angioplastia e colocação de stent. O presente estudo contou com um banco de dados centralizado, contendo um perfil demográfico, dados clínicos, ECGs, tratamentos, intervalos de tempo e eventos hospitalares. Portanto, todos os desfechos relevantes foram sistematicamente registrados e as taxas de mortalidade foram analisadas por observadores independentes.

### Definições de apresentações clínicas

A apresentação clínica de sintomas isquêmicos agudos foi relatada pelos pacientes, e equipes treinadas analisaram os dados durante o período de internação hospitalar.

1. Dor torácica típica: dor torácica opressiva à esquerda, que pode se irradiar para o membro superior esquerdo, de grande intensidade e prolongada (mais de 20 minutos), que não melhorou ou teve alívio apenas parcial com repouso ou nitrato sublingual. A irradiação para mandíbula, membro superior direito, dorso, ombros e epigástrio também foi considerada para essa apresentação. Esse grupo incluiu pacientes com apresentação concomitante de dispneia ou episódio sincopal.
2. Dor atípica: dor no quadrante superior direito ou região epigástrica do abdômen, dorsal, região mandibular ou outra região não torácica, referida como pontada, queimação de intensidade variável, duração prolongada (maior que 20 minutos). Incluídos nesse grupo estavam os pacientes com apresentação concomitante de dispneia ou episódios sincopais.
3. Dispneia: foram incluídos neste grupo os pacientes que não relataram dor torácica, mas que se queixaram de cansaço agudo ou piora desse sintoma nas últimas horas. Foi considerada a experiência subjetiva de desconforto respiratório, composta por sensações qualitativamente diferentes e com intensidade variável.
4. Síncope: foram considerados os pacientes que não relataram dor torácica, mas sim desmaio ou perda súbita e transitória da consciência ou qualquer piora nas últimas horas.

### Tempos pivotais medidos

1. Intervalo de tempo entre o início da dor torácica persistente, ou outra queixa representativa de sintomas isquêmicos, e a chegada do paciente à unidade de saúde;
2. Intervalo de tempo entre chegada à unidade de saúde e trombólise;
3. Intervalo de tempo entre a trombólise e a angiografia coronária.

### Escores de previsão

### Preditores de risco usados durante a primeira consulta médica

1. Classificação Killip-Kimball (KK);^6^
2. TIMI-Risk;^7^
3. Escore GRACE^8^

### Variáveis angiográficas

Cardiologistas intervencionistas experientes realizaram análises angiográficas pelo escore de fluxo [(TIMI- flow), perfusão coronária epicárdica];^9^ e [Myocardial Blush Grade (MBG), perfusão miocárdica no nível do tecido];^10^ obtendo-se TIMI-flow e MBG antes e pós-intervenção percutânea, quando aplicáveis (chamados de escores iniciais e finais). Complicações inerentes ao procedimento também foram relatadas. A estratégia do procedimento (aspiração do trombo, dilatação do balão, seleção do stent e regime de anticoagulação) foi deixada a critério do operador.

### Análise estatística

Este estudo buscou alcançar uma coleta de dados prospectiva e consecutiva de uma grande população, em que práticas médicas padrão atuais são aplicadas em uma rede organizada. As variáveis contínuas foram expressas como média ± desvio padrão (DP) ou mediana e faixa interquartil [FIQ (25º - 75º percentis)], de acordo coma normalidade dos dados. Para avaliar as suposições de normalidade, usamos o teste D’Agostino-Pearson, desenvolvido para avaliar uma amostragem grande, e a confirmação foi feita pela inspeção visual de gráficos de dispersão. Variáveis categóricas foram descritas como frequências absolutas e relativas, e examinadas pelo teste qui-quadrado de Pearson. Para comparações de variáveis numéricas entre grupos, usamos o teste t de Student não pareado ou o teste U de Mann-Whitney quando a distribuição não-Gaussiana foi considerada. As análises de variância simples pelo teste “t”, ou de seus equivalentes não paramétricos foram realizadas para a observação da distribuição e da homocedasticidade dos valores. Para comparar proporções entre os grupos, o teste χ^2^ (qui-quadrado) foi usado para amostras independentes. Para presumir uma igualdade de variância entre os grupos, os ajustes foram feitos usando-se o teste de Levene. O risco relativo foi determinado pela razão entre os portadores de determinada variável e os não portadores. Para analisar a relação de algumas variáveis categóricas e os desfechos, elas foram transformadas em dicotômicas. Portanto, o teste de proporção (qui-quadrado) foi usado para observar a independência entre as univariadas para obter as razões de chance (RC) em um modelo de correlação entre univariadas potencialmente preditivas e os desfechos. Na estatística multivariada verificamos as relações de múltiplas variáveis, apenas para as com significância na entrada (variáveis com p <0,10), para a observação de seu grau de independência. Utilizamos o modelo de regressão logística binária, pela técnica de máxima verossimilhança, em que a variável dependente era dicotômica e as variáveis preditoras inseridas pelo modelo *stepwise*, considerando a ausência de colinearidade pelo índice VIF (fator de inflação da variância), com a qualidade do ajuste avaliada pelo diagrama de Hosmer-Lemeshow. As variáveis preditoras foram analisadas simultaneamente, de forma que o efeito de cada variável foi ajustado para ter um efeito nas demais. Esse modelo de regressão adiciona sistematicamente a variável mais significativa ou remove a variável menos significativa durante cada etapa. O índice **α** de Crombach-padronizado foi usado para calcular a confiabilidade dos escores angiográficos TIMI-flow e MBG estimados pelos operadores médicos.

As curvas de característica de operação do receptor (ROC) foram construídas para determinar a sensibilidade e a especificidade dos escores de predição de desfechos hospitalares. A forma da curva ROC e a área sob a curva (AUC) ajudaram a estimar o nível de poder discriminativo de um teste. Um teste diagnóstico perfeito tem uma AUC de 1,0, enquanto um teste não discriminatório tem uma área de 0,5. Outras análises também foram aplicadas, considerando as razões de probabilidade na previsão de eventos. Portanto, com base nas razões de probabilidade, a razão de chance diagnóstica (RCD), uma métrica global de precisão diagnóstica, foi calculada, ou seja, a razão de chance de positividade em sujeitos com o desfecho para a chance em sujeitos sem o desfecho. Consideramos o valor de p<0,05 como estatisticamente significativo, em testes bicaudais. As análises foram realizadas usando SPSS-versão-20 (IBM-SPSS Statistics, EUA)^®^.

## Resultados

### Características clínicas e epidemiológicas

A Tabela 1 mostra que os pacientes tinham uma mediana (FIQ) de 58 (50-65) anos de idade, e aproximadamente 70% eram homens. A maioria dos pacientes era hipertenso e fumante, e aproximadamente um terço deles tinha diabetes. Uma pequena proporção dos pacientes teve eventos anteriores, tais como infarto do miocárdio, revascularização cirúrgica ou percutânea, e acidente vascular cerebral prévio. O grupo mais velho tinha ≥60 anos de idade. Os preditores de risco incluíam dados sobre o histórico médico do paciente e os fatores de risco, que foram analisados durante a admissão hospitalar. Este estudo também apresenta dados sobre variáveis hemodinâmicas obtidas na emergência, tais como dados laboratoriais (necrose e marcadores bioquímicos) (Tabela 2).

**Tabela 1:** Características da linha de base da coorte estudada

Variáveis	
Epidemiológicas	Medidas
Idade, md (FIQ) - anos	58 (50-65)
Mulheres: md (FIQ) - anos	60 (52-68)
Homens: md (FIQ) - anos	57 (49-64)
Idosos (≥60 anos), n (%)	998 (43,6)
Sexo; n (%)	Masculino: 1607 (70,2)
	Feminino: 683 (29,8)
Hábitos - Vícios	Medidas
Tabagismo, n (%)	1472 (64,3)
Alcoolismo, n (%)	304 (13,3)
Drogas ilícitas, n (%)	97 (4.2)
Clínicas	
Obesidade n (%)	481 (21,0)
IMC, Kg/m²; md (FIQ)	26 (23,8-29,3)
Hipertensão arterial, n (%)	1405 (61,4)
Diabetes mellitus, n (%)	661 (28,9)
Dislipidemia, n (%)	1133 (49,5)
Hipotireoidismo, n (%)	145 (6,3)
Doença renal crônica, n (%)	187 (8,2)
Doença arterial periférica, n (%)	117 (5,1)
Acidente vascular cerebral prévio, n (%)	99 (4.3)
Síndrome coronariana crônica	Medidas
Infarto do miocárdio prévio, n (%)	242 (10,6)
Angioplastia coronária prévia, n (%)	129 (5,6)
Revascularização miocárdica cirúrgica prévia, n (%)	45 (2)

Dados sobre histórico médico e comorbidades foram derivados de entrevistas com médicos. As informações demográficas e os perfis de fatores de risco foram relatados por pacientes, e uma equipe treinada analisou esses dados durante a hospitalização. Dados são expressos como mediana (md) e faixa interquartil (FIQ), e variáveis categóricas foram expressas como frequência (%). Para a comparação entre sexos, o teste não paramétrico de Mann-Whitney foi usado. Doença renal crônica (DRC) foi estimada pela equação de Modificação da dieta em doença renal (MDRD) e definida quando a taxa de filtração glomerular (TFGe) estimada <60 mL/min/1,73 m2; obesidade quando IMC ≥30; hipertensão definida pelo uso de medicamentos anti-hipertensivos ou pressão arterial sistólica ≥140 mmHg e/ou pressão arterial diastólica ≥90 mmHg. Tabagismo foi definido tanto para ex-fumantes como para fumantes ativos; dislipidemia foi definida pelo uso de medicamentos específicos ou colesterol total ≥200 mg/dL, ou triglicérides ≥150 mg/dL; diabetes foi definida como tratamento específico ou hemoglobina glicada (HbA1c) ≥6,5%. IMC: Índice de massa corporal.

**Tabela 2:** Características clínicas e hemodinâmicas, escores de predição e tempos pivotais foram obtidos durante a primeira consulta médica e durante o período de internação hospitalar

Variáveis obtidas durante a primeira consulta	Medidas
**Variáveis hemodinâmicas**	
Pressão arterial sistólica, mmHg; md (FIQ)	130 (115-150)
Pressão arterial diastólica, mmHg; md (FIQ)	80 (70-93)
Frequência cardíaca, md (FIQ)	76 (66-90)
Classificação Killip-Kimball, n (%)	Killip-Kimball – I: 1670 (72,0)
	Killip-Kimball – II: 362 (15,8)
	Killip-Kimball – III: 52 (2.3)
	Killip-Kimball – IV: 203 (8,0)
**Apresentação clínica** (sintoma principal), n (%)	Dor típica: 1939 (88,5)
	Dor atípica: 166 (7,6)
	Dispneia: 38 (1.7)
	Síncope: 26 (1.2)
	* *Alguns pacientes (4%) com mais de um sintoma relatado*
**Escores de risco**	
TIMI-Risk, (0-14); md (FIQ)	3 (2-5)
Escore GRACE, (0-14); md (FIQ)	135 (115-160)
**Tempos pivotais**	
Tempo dor-unidade de saúde, min; md (FIQ)	120 (60-220)
Tempo dor-agulha, min; md (FIQ)	222 (140-345)
Tempo porta-agulha, min; md (FIQ)	71 (42-135)
Tempo lise-angiografia, horas, md (FIQ)	12 (5,67-23)
**Variáveis obtidas durante a internação hospitalar**	Medidas
**Biomarcadores de necrose**	
Troponina inicial, mg/L; md (FIQ)	2655 (538-7967)
Troponina máxima, mg/L; md (FIQ)	4718 (1481-9842)
**Variáveis laboratoriais**	
Hemoglobina/hematócritos, g/dL/%; m ± DP	14,37 ± 2,09 /42,92 ± 12,56
Glicemia do sangue/Hemoglobina glicada, mg/dL / %; md (FIQ)	122 (102-160) / 6 (5,6-6,8)
Colesterol total, mg/dL; md (FIQ)	191 (157-225)
HDL-C, mg/dL; md (FIQ)	37 (25-46)
LDL-C, mg/dL; md (FIQ)	110 (60-142)
Triglicérides, mg/dL; md (FIQ)	118 (77-175)
*AST, u/L; md (FIQ)	144 (63-280)
†ALT, u/L; md (FIQ)	43 (27-72)
Creatinina, mg/dL; md (FIQ)	0,9 (0,74-1,10)
Taxa de filtração glomerular estimada, (MDRD); md (FIQ)	85 (64-107)

Notas: Dados são expressos como mediana (md) e faixa interquartil (FIQ) ou média e desvio padrão (m ± dp), e variáveis categóricas foram expressas como frequência (%). Métricas de tempo são expressas em minutos (min). Taxa de filtração glomerular por Modificação da dieta em doença renal (MDRD)

* AST: aspartato aminotransferase

† ALT: alanina aminotransferase.

### Escores de predição, apresentação clínica e tempos pivotais

Na análise dos sintomas isquêmicos, a maioria dos pacientes relataram dor torácica definida como típica, que pode ou não estar associada a dispneia e síncope. Angina antes do evento estava presente em 28%, que era mais prevalente em pacientes com infarto do miocárdio prévio. As mulheres apresentaram uma alta frequência de sintomas atípicos, tais como dispneia e síncope, conforme mostrado na Tabela 3.

**Tabela 3:** Variáveis associadas ao tipo de apresentação clínica em um modelo univariado e após ajustes multivariados em regressão logística multinomial

Modelo sem ajustes	Dor típica	Dor atípica	Dispneia	Síncope
Variáveis	**Razão de chance** (IC 95%), p-valor	**Razão de chance** (IC 95%), p-valor	**Razão de chance** (IC 95%), p-valor	**Razão de chance** (IC 95%), p-valor
	n = 1939	n = 166	n = 38	n = 26
Masculino	0,95 (0,71-1,27), p=0,74	1,05 (0,79-1,40), p=0,73	**0,51** (0,27-0,96), p=0,026	**0,39** (0,18-0,83), p=0,018
Obesidade	1,07 (0,78-1,50), p=0,65	0,92 (0,67-1,28), p=0,65	**0,30** (0,09-0,98), p=0,02	0,29 (0,07-1,26), p=0,09
Alcoolismo	0,80 (0,56-1,15), p=0,24	1,24 (0,86-1,78), p=0,24	1,15 (0,48-2,77), p=0,44	1,49 (0,56-3,97), p=0,39
Hipertensão	0,96 (0,73-1,26), p=0,78	1,03 (0,79-1,35), p=0,79	1,31 (0,67-2,56), p=0,26	0,67 (0,31-1,44), p =0,32
Dislipidemia	**1,39** (1,07-1,81), p=0,013	**0,71** (0,55-0,93), p=0,013	1,25 (0,66-2,35), p=0,52	**0,35** (0,15-0,84), p =0,019
Infarto do miocárdio prévio	1,05 (0,68-1,61), p=0,81	0,95 (0,62-1,46), p=0,82	**3,31** (1,63-6,72), p=0,002	0,76 (0,16-2,86), p =0,59
Acidente vascular cerebral prévio	0,75 (0,42-1,34), p=0,34	1,32 (0,74-2,37), p=0,34	1,82 (0,55-6,00), p=0,24	0,85 (0,11-6,32), p =0,87
Doença arterial periférica	0,78 (0,45-1,35), p=0,30	1,27 (0,74-2,20), p=0,38	1,52 (0,46-5,00), p=0,45	0,71 (0,09-5,29), p =0,74
Doença renal crônica	**0,53** (0,35-0,79), p=0,002	**1,88** (1,26-2,80), p=0,002	**4,47** (2,19-9,10), p<0,001	1,97 (0,67-5,77), p =0,27
Tabagismo	1,17 (0,90-1,53), p=0,23	0,85 (0,65-1,11), p=0,23	1,15 (0,59-2,25), p=0,74	0,59 (0,27-1,27), p =0,22
Diabetes	0,95 (0,72-1,27), p=0,76	1,04 (0,78-1,39), p=0,75	**2,26** (1,21-4,24), p=0,013	0,70 (0,28-1,74), p =0,52
Idosos	0,87 (0,67-1,13), p=0,32	1,14 (0,88-1,48), p=0,32	1,77 (0,94-3,33), p=0,07	0,76 (0,34-1,66) p =0,56
**Modelo ajustado**	**Dor típica**	**Dor atípica**	**Dispneia**	**Síncope**
Variável	**Razão de chance** (IC 95%), p-valor	**Razão de chance** (IC 95%), p-valor	**Razão de chance** (IC 95%), p-valor	**Razão de chance** (IC 95%), p-valor
Homens	NA	NA	**0,51** (0,26-0,97), p=0,04	**0,32** (0,15-0,70), p=0,005
Obesidade	NA	NA	0,29 (0,08-0,95), p =0,04	NA
Dislipidemia	**1,44** (1,10-1,87), p=0,007	**0,69** (0,53-0,90), p =0,007	NA	**0,36** (0,15-0,87), p=0,02
Infarto do miocárdio prévio	NA	NA	2,68 (1,28-5,58), p=0,008	NA
Doença renal crônica	**0,50** (0,34-0,75), p=0,001	**1,97** (1,32-2,94), p=0,001	**3,33** (1,59-6,98), p=0,001	NA
Diabetes mellitus	NA	NA	**1,93** (1,01-3,71), p=0,04	NA

Notas: Os dados são expressos para (RC; IC 95%, p-valor). Na análise multivariada, as variáveis preditoras foram analisadas simultaneamente, de forma que o efeito de cada variável foi ajustado para ter um efeito nas demais. Negrito indica significância estatística. Doença renal crônica (DRC) foi estimada pela Modificação da dieta em doença renal (MDRD); idosos: idade ≥60 anos. Negrito indica significância estatística. NA: não aplicável.

**Figura 1:**
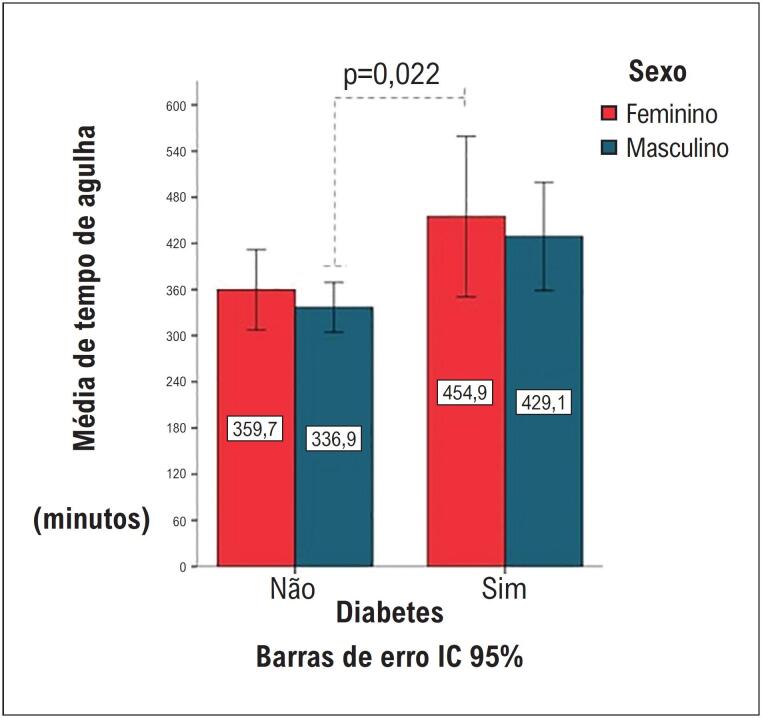
Métricas de tempo relacionadas a sexo e presença ou ausência de diabetes. Nota. Tempo dor-agulha expresso como média (minutos). * P-valor significativo ao se comparar homens não diabéticos com mulheres diabéticas.

As mulheres apresentaram uma demora entre a chegada do paciente na unidade de emergência e o início do tratamento: [mulheres (2 horas e 17 minutos) vs. homens (1 hora e 58 minutos), p=0,021]. O tempo estratificado (≥240 minutos) para receber o tratamento foi favorável aos homens: [RC 0,73; IC 95% (0,55-0,98), p=0,03]. Outra constatação relevante foi o intervalo mais longo entre o aparecimento dos sintomas e a procura de tratamento médico entre as mulheres: [mulheres: (3 horas e 14 minutos) vs. homens (2 horas e 48 minutos), p=0,008]. Mulheres diabéticas apresentaram maior tempo entre o início dos sintomas até a trombólise, incluindo, nesse período, a chegada à unidade de pronto socorro no início do tratamento (intervalo entre dor e trombólise), especialmente quando comparadas aos homens não diabéticos (Figura 1).

A maioria dos pacientes estava em uma classe funcional baixa de acordo com o escore KK: [I (73%), II (16,3%), III (2,2%), e IV (8,6%)]; perfil de baixo risco no escore de previsão TIMI-Risk: [3, FIQ (2-5)]; e escore GRACE: [136, FIQ (117-161)]. Aproximadamente 24% dos pacientes foram encaminhados para angioplastia de resgate, conforme avaliação da equipe médica local, por não atingirem os critérios para uma terapia de reperfusão bem-sucedida.

### Achados angiográficos (artéria culpada)

1) artéria descendente anterior esquerda (ADAE): 46,3%; 2) artéria coronária direita (ACD): 32,1%; 3) artéria circunflexa esquerda (ACX): 6,1%; 4) artéria coronária esquerda principal (ACEP): 0,4%; 5) artéria descendente posterior (ADP): 0,3%; 6) tronco ventricular posterior (VP): 0,9%; 7) ramificação marginal esquerda: 1,0%; 8) ramificação diagonal: 0,7%; 9) artéria não identificada: 12,1% A análise de subgrupo não encontrou associações significativas entre a artéria culpada e fatores de risco prévios, nem com a apresentação clínica inicial de sintomas isquêmicos.

Os escores TIMI-flow e MBG (iniciais e finais) foram registrados, classificados de 0 a 3: [TIMI-flow inicial: (3)] e [MBG inicial: (3)] em aproximadamente 60% e 42%, respectivamente. Quando esses escores pós-procedimento percutâneos foram analisados, os índices de TIMI-flow final: (3) e de MGB final: (3) eram 78% e 58%, respectivamente, com um alto nível de confiabilidade. O **α** de Crombach = 0,88.

### Desfechos associados a complicações no laboratório de hemodinâmica e pelo evento índice

A duração média da internação hospitalar foi de 2,0 ± 1,3 dias, da admissão no hospital terciário até a alta ou transferência para um hospital de contrarreferência para continuar o tratamento. Um período de internação mais longo foi observado no grupo dos idosos: [1,9 dias (não idosos) vs. 2,3 dias (idosos), p=0,004] e notadamente no grupo de pacientes que precisaram receber transfusões de material sanguíneo devido a sangramento: [1,9 dias (sem sangramento) vs. 3,4 dias (sangramento importante), p=0,004].

O presente estudo registrou a frequência das complicações durante o procedimento angiográfico, bem como aqueles observados durante o período de hospitalização. Essas complicações incluíram eventos importantes, tais como dissecção ou ruptura da coronária, entre outros, como complicações clínicas, arritmias e sangramento no local na incisão. Embora infrequentes, sangramentos importantes eram independentemente associados à mortalidade hospitalar. Índices de sangramento mais altos foram observados no grupo dos idosos: [RC: 1,86; IC 95% (1,04-3,16), p=0,023].

### Preditores e variáveis associadas às taxas de mortalidade

A taxa de mortalidade hospitalar era 5,6%, com 128 mortes, das quais 23 (17,9%) ocorreram no laboratório de hemodinâmica, com instabilidade elétrica ou complicações mecânicas sendo as causas mais prevalentes, e com uma incidência mais alta ocorrendo no grupo de resgate (11,5% vs. 2,4%). Em relação às características epidemiológicas e fatores de risco, uma distribuição similar das variáveis analisadas foi observada em pacientes sobreviventes, em comparação com o que morreram durante o período de internação, exceto pelo escore GRACE, que apresentou valores mais altos nos pacientes que morreram [134 (115-157) vs. 202 (155-233), p<0,001]. O escore GRACE (escore mediano 136) apresentou boa sensibilidade mais especificidade baixa [sensibilidade: 0,86%; especificidade: 0,53%]. O escore de previsão TIMI-Risk (escore mediano 3) apresentou os seguintes resultados: sensibilidade: 0,87%; especificidade: 0,57% (Tabela 4).

**Tabela 4:** Variáveis clínicas e epidemiológicas entre os grupos de “sobreviventes” e “mortes”

		Grupo de sobreviventes	Grupo de mortes	p-valor
**Variáveis**		2162 (94,4%)	128 (5,6%)	
**Epidemiológicas**				
Idade, anos:		58 (50-66)	56 (48-65)	**0,047**
Sexo masculino:		Homens: 71,1%	Homens: 53,2%	**0,02**
**Escores de risco**				
Killip-Kimball, (%)		Killip-Kimball – I: 75,6%	Killip-Kimball – I: 20,6%	0,09
		Killip-Kimball – II: 16,4%	Killip-Kimball – II: 7,9%	0,42
		Killip-Kimball – III: 2,2%	Killip-Kimball – III: 4,8%	0,96
		Killip-Kimball – IV: 5,7%	Killip-Kimball – IV: 66,5%	0,08
TIMI-Risk:		3 (2-5)	6 (5-8,2)	0,26
Escore GRACE:		134 (115-157)	202 (155-233)	**p <0,001**
**Variáveis de ECG**				
	Parede do ECG	parede anterior	parede anterior	0,68
		parede inferior	parede inferior	0,58
		parede lateral	parede lateral	0,45
**Variáveis hemodinâmicas**				
Artéria relacionada ao infarto		ADA: 45,5%	ADA: 38,9%	0,84
		ACD: 32,5%	ACD: 36,5%	
		ACX: 6%	ACX: 10,3%	
		Outros: 22,4%	Outros: 14,3%	
†Fração de ejeção ventricular esquerda (FEVE):		50 (40-59)	49 (40-60)	0,17
TIMI-flow inicial		TIMI-0: 19,6%	TIMI-0: 36,8%	0,97
		TIMI-1: 3,3%	TIMI-1: 11,3%	
		TIMI-2: 15,4%	TIMI-2: 17%	
		TIMI-3: 61,7%	TIMI-3: 34,9%	
TIMI-flow final		TIMI-0: 4,6%	TIMI-0: 19,8%	0,10
		TIMI-1: 1,2%	TIMI-1: 4,7%	
		TIMI-2: 14,1%	TIMI-2: 24,5%	
		TIMI-3: 80,1%	TIMI-3: 50,9%	
Grau Blush miocárdico inicial:		Blush-0: 40,8%	Blush-0: 71,7%	0,77
		Blush-1: 3,8%	Blush-1: 2,8%	
		Blush-2: 2,6%	Blush-2: 1,9%	
		Blush-3: 52,8%	Blush-3: 23,6%	
Grau Blush miocárdico final:		Blush-0: 24,7%	Blush-0: 61%	0,39
		Blush-1: 8,8%	Blush-1: 9,5%	
		Blush-2: 6,1%	Blush-2: 5,7%	
		Blush-3: 60,4%	Blush-3: 23,8%	
**Variáveis laboratoriais**				
Troponina inicial (na linha de base)		2704 (618-7889)	3413 (280-11506)	0,83
Troponina máxima:		4820 (1661-9796)	7925 (1145-1774)	0,80
^*^TFGe, (MDRD):		86 (67-107)	91 (66-111)	0,62
Hemoglobina:		14,5 (13,3-15,7)	13,7 (12,5-15,1)	0,05
Hematócritos:		42,9 (39,6-46,2)	41 (37,9-45,5)	0,14
**Tempos pivotais**				
Tempo dor-agulha, (min):		220 (140-345)	245 (150-516)	0,08
Tempo porta-agulha, (min):		75 (45-135)	78,5 (45-163,7)	0,12
‡Tempo lise-cateterismo, (horas):		11 (5-22)	11 (5-21,7)	0,43

Notas: Dados são expressos como mediana (md) e faixa interquartil (FIQ), e variáveis categóricas foram expressas como frequência (%). O teste χ² (qui-quadrado) foi usado para as amostras independentes. TFGe: Taxa de filtração glomerular estimada por Modificação da dieta em doença renal (MDRD).

† FEVE: Fração de ejeção ventricular esquerda.

‡ Lise-CATE (fibrinólise-cateterismo); ECG: eletrocardiograma. Negrito indica significância estatística.

A classificação funcional KK demonstrou bom desempenho na previsão da mortalidade hospitalar: AUC: [0,77 IC 95% (0,73-0,81), p<0,001] no grupo com um escore ≥II, demonstrando melhor precisão que os escores angiográficos de reperfusão: TIMI-Flow (3) e MBG (3), AUC: [0,69; IC 95% (0,64-0,75), p<0,001], bem como um melhor desempenho quando os pacientes foram estratificados de acordo com a fração de ejeção ventricular esquerda, AUC: [0.52; 95% CI (0,47-0,58), p=0,34].

**Figura 2:**
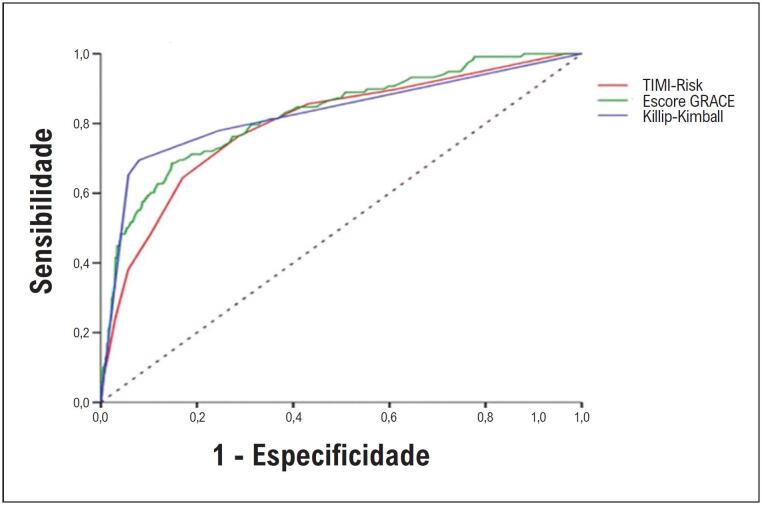
Escores de previsão para mortalidade hospitalar. Nota. Estatísticas C, ROC e AUC. TIMI-Risk, AUC: [0,79; IC 95% (0,75-0,84), p<0,001]; Escore GRACE, AUC: [0,82; IC 95% (0,78-0,86), p<0,001]; Killip-Kimball, AUC: [0,82; IC 95% (0,78-0,87), p<0,001].

Nosso estudo teve o cuidado de avaliar o desempenho dos escores de previsão: TIMI-Risk, AUC: [0,79; IC 95% (0,75-0,84)], p<0,001; GRACE, AUC: [0,82; IC 95% (0,78-0,86), p<0,001]; AUC de Killip-Kimball AUC: [0,82; IC 95% (0,78-0,87), p<0,001], (Figura 2). Para as categorias de Killip-Kimball, os seguintes escores foram obtidos: razão de probabilidade positiva: 3,76; razão de probabilidade negativa: 0,33; e RCD (Razão de chance diagnóstica: 11,39 para os índices de previsão de mortalidade hospitalar, definidos como a probabilidade dos pacientes na classe funcional ≥ II (II, III, IV) que morreram, em relação à probabilidade dos pacientes do grupo funcional ≥ II que sobreviveram.

**Figura 3:**
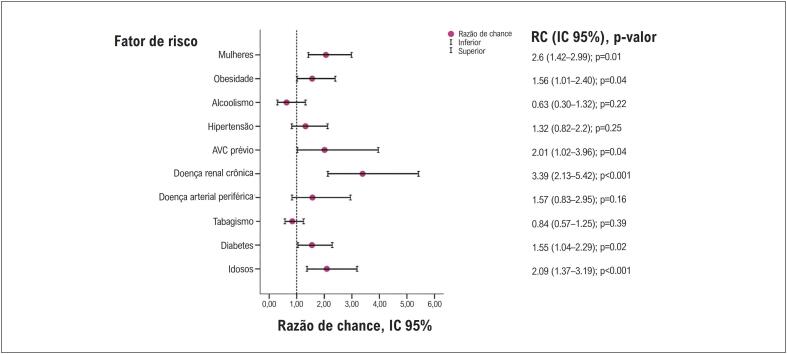
Preditores de mortalidade hospitalar Nota. Variáveis de previsão em um modelo de regressão logística binária, com RC-IC 95%, e p-valor.

Em um modelo de regressão logística com análises de covariância, obesidade, sexo feminino, pacientes com diabetes mellitus, insuficiência renal crônica, acidente vascular cerebral prévio e a idade estavam associados aos índices mais altos de eventos fatais (Figura 3). O ajuste do modelo apresentou um bom desempenho preditivo.

## Discussão

Mesmo com a existência de várias terapias eficazes, as informações qualitativas ainda não estão disponíveis para estratificação, especialmente na estratégia fármaco-invasiva, em que a avaliação de saúde inicial parece estar proximamente relacionada ao prognóstico. Há muitas abordagens para a estratificação de risco. Algumas são muito complexas e caras, mas procedimentos simples também podem ser eficazes. Sob essa perspectiva, o presente estudo tentou oferecer dados epidemiológicos sobre as várias formas de sintomas isquêmicos agudos, bem como demonstrar a aplicabilidade de alguns escores de previsão em pacientes que receberam cuidados médicos principalmente nas unidades de saúde básica, e foram encaminhados a um hospital universitário terciário para estudos angiográficos e procedimentos invasivos.

Nem todas as síndromes coronárias agudas apresentam sinais ou sintomas clássicos, tais como dor pré-cordial ou retroesternal típica, o que atrasa o diagnóstico e as abordagens terapêuticas e afeta o prognóstico. Portanto, há grande interesse nos principais determinantes de mortalidade e complicações de curto prazo após um infarto agudo do miocárdio.^11^ Nos últimos anos, grandes avanços no diagnóstico e no tratamento contribuíram para a redução da mortalidade devido a doença cardíaca coronária. Na verdade, foram feitos esforços para se alcançar a prevenção primária mais ativa, com um controle melhor dos fatores de risco, e recursos farmacológicos. Entretanto, a prevenção e o tratamento de doenças cardiovasculares precisam estar amplamente disponíveis e aplicados sem distinção.

### Apresentação clínica e eventos em subgrupos

Há evidências de diferenças entre os sexos na aplicação dos avanços tecnológicos.^12–15^ Em nossa análise, os sintomas referidos como dispneia e episódios sincopais foram significativamente mais prevalentes em mulheres, e eram particularmente relevantes para esse grupo, apresentando taxas de mortalidade mais altas. Vários mecanismos multifatoriais foram propostos para a maior mortalidade cardíaca entre mulheres. As explicações geralmente incluem um diâmetro menor da coronária em mulheres, fluxo colateral baixo, predisposição a erosão de placas e embolização distal, e outras características fenotípicas de placas ateroscleróticas.^16–18^ As mulheres geralmente apresentam eventos aproximadamente uma década depois dos homens, especialmente na pós-menopausa, possivelmente devido à diminuição do estrogênio e à perda de efeitos protetivos, com a consequente piora dos fatores de risco, ganho de peso, e resistência à insulina.^19–21^ Em nossa coorte, a demora em reconhecer os sintomas, possivelmente devido a sua aparência atípica, teve um impacto em métricas temporais para trombólise, tais como os registrados nos grupos de mulheres e diabéticos.

Diabetes é um fator de risco potencial em mulheres. Uma meta-análise para estimar o risco relativo de doença cardíaca coronária fatal associada ao diabetes, envolvendo aproximadamente 450.000 pacientes, revelou um risco relativo 50% mais alto nas mulheres.^22^ O risco coronário mais alto associado ao diabetes em mulheres pode refletir um viés de tratamento favorável aos homens. Estudos mostram que homens com diabetes ou doença cardiovascular estabelecida têm maior probabilidade de receber tratamento com antiagregantes plaquetários, estatinas ou medicamentos anti-hipertensivos que as mulheres.^23,24^ Além disso, há relatórios de má adesão às recomendações de diretrizes entre as mulheres, tais como um tempo mais longo entre a chegada no hospital e a colocação do balão.^25–28^ Também observou-se que os homens receberam a terapia fibrinolítica mais cedo, possivelmente devido à apresentação mais clara de sintomas isquêmicos.

A aparência clínica, definida acima como dispneia, era mais prevalente em pacientes com infartos anteriores, possivelmente devido a comprometimento miocárdico adicional. O relato de dispneia também foi feito por pacientes com diabetes. Isso pode ter tido um impacto significativo na taxa de mortalidade devido à ausência de sintomas típicos ou sinais de alerta.

### Escores de risco

Embora não exista um modelo de estratificação ideal, ele deve conter as seguintes características: facilidade de implementação, objetividade, precisão e uso disseminado. Killip-Kimball, um método de classificação funcional aplicado durante a primeira consulta médica, foi um preditor importante de desfechos fatais durante o período de internação hospitalar, com um bom valor preditivo negativo. O índice de gravidade da insuficiência cardíaca em pacientes com infarto agudo do miocárdio foi proposto por Thomas Killip e John Kimball numa tentativa de medir o risco de eventos hospitalares e possível benefício da gestão específica do tratamento médico oferecido em unidades de tratamento coronário. Nossa análise destaca o uso clínico de um exame físico como uma ferramenta simples, sem requisitos tecnológicos sofisticados para identificar os sinais de insuficiência cardíaca na admissão hospitalar, que desempenhou um papel prognóstico relevante nas taxas de mortalidade durante a internação, uma vez que as proporções de mortes e na distribuição de dados de sobrevida foram significativamente diferentes dentro da classe >I de Killip-Kimball.

### Taxas de mortalidade hospitalar

A taxa de mortalidade, incluindo eventos durante o procedimento angiográfico e os relacionados ao evento índice, estava associada a complicações mecânicas e distúrbios elétricos graves e irreversíveis. Relatou-se que a demora no tempo de recanalização está associada a um comprometimento maior da função ventricular, distúrbios de microcirculação e taxas de mortalidade mais altas.^29^ É interessante notar que nosso estudo não identificou associação entre as taxas de mortalidade entre pacientes no grupo com fração de ejeção ventricular esquerda (FEVE) mais baixa nem com os escores angiográficos. De fato, a lesão culpada totalmente ocluída (TIMI-flow-0) não demonstrou estar associada às maiores taxas de mortalidade hospitalar após o IAMCSST tratado com TNK, em comparação com aqueles que apresentavam TIMI-flow ≥1. Além disso, as arritmias ventriculares malignas podem aparecer mais cedo nos processos isquêmicos e continuar a ser uma causa esperada de morte em infartos do miocárdio.^30,31^ Em nossa coorte, as taxas de mortalidade hospitalar foram mais altas entre os pacientes que apresentaram arritmias ventriculares malignas do que os pacientes que não apresentaram arritmias ventriculares, definidas como taquicardia ou fibrilação. Entretanto, devido à característica observacional de nosso estudo, várias dificuldades foram encontradas na caracterização das arritmias ventriculares, especialmente no período pós-angioplastia, já que a ocorrência desses eventos foi era difícil de prever. Portanto, pode-se especular que distúrbios elétricos graves podem ser um marcador forte de desfechos hospitalares, apesar do sucesso da intervenção coronária percutânea e seus respectivos escores angiográficos, e também podem não estar correlacionados a FEVE. Presume-se que esse seja um marcador melhor quando aplicado a desfechos medidos no médio e no longo prazo.

Alguns subgrupos de interesse especial foram examinados em sua associação com taxas de mortalidade, tais como o grupo das mulheres, os pacientes obesos, os diabéticos e os idosos. Nesse sentido, após o infarto do miocárdio, as mulheres parecem estar em maior risco de um novo infarto e morte, o que pode ser parcialmente explicado pela idade mais avançada, como observado em nosso estudo.

Em contraste, a doença renal crônica (DRC) representa um fator de risco independente para o desenvolvimento de doença cardíaca isquêmica, aumentando a mortalidade com o avanço do comprometimento renal. Lesões renais prévias ou resultantes do infarto estão associadas a desfechos piores.^32,33^ É provável que haja uma relação recíproca entre o processo aterosclerótico coronário e a função renal, e a presença de doença coronária esteja associada a uma piora da função renal. DRC e doença cardiovascular estão intimamente relacionadas, e a presença de uma condição sinergicamente afeta o prognóstico da outra.^34^ Nossos dados expressam as taxas de mortalidade mais altas para esses pacientes.

Pacientes que sofreram um acidente vascular cerebral prévio também apresentaram taxas mais altas de eventos fatais, destacando a necessidade de prevenção e tratamento adequado para esse subgrupo.^35,36^ O mesmo conceito pode ser aplicável aos idosos, possivelmente devido a maior fragilidade biológica nesse grupo. Os mecanismos para essa relação parecem ser múltiplos, envolvendo características anatômicas, bioquímicas e imunológicas, ou mesmo a exposição mais longa a fatores de risco clássicos. Outro achado em nossa coorte foi a taxa de mortalidade mais alta nos pacientes com diabetes mellitus, o que está de acordo com estudos prévios.^37–39^ O diabetes mellitus frequentemente está associado a múltiplos mecanismos de doença cardiovascular, tais como obesidade, hipertensão, insuficiência renal, inflamação subclínica, disfunção endotelial e comprometimento microvascular. No entanto, não foram encontradas diferenças significativas nos escores angiográficos de reperfusão MBG ou TIMI-flow na comparação de grupos de pacientes com e sem diabetes.

### Pontos fortes e limitações

O presente estudo tem limitações. Primeiramente, foi um estudo observacional, com correção de fatores de confusão medidos ou conhecidos. Portanto, não podemos concluir que as associações observadas são causais. Além disso, nossos registros incluem apenas pacientes que foram submetidos a intervenção fármaco-invasiva, desconsiderando aqueles que foram encaminhados para tratamento percutâneo primário ou que tinham contraindicações formais para fibrinólise. Considerando que o estudo foi realizado em um único centro universitário, esses padrões de prática e resultados devem ser interpretados com cuidado. Outra limitação de nosso estudo foi a análise dos desfechos apenas para o período de internação hospitalar. Em um sentido importante, nossa validação interna indicou que o ajuste do modelo era bom e os modelos de previsão diagnóstica tiveram um bom desempenho para fazer a previsão do prognóstico independentemente.

## Conclusões

Nossos dados revelaram taxas de mortalidade hospitalar mais altas em mulheres, em pacientes com diabetes mellitus, obesidade, DRC e acidentes vasculares prévios, bem como em idosos. A disparidade relacionada a sexo persiste nas mulheres, com demoras no reconhecimento dos sintomas de isquemia, e o início imediato de terapia fibrinolítica, levando a priores resultados clínicos. A aplicabilidade do escore de Killip-Kimball para prever eventos fatais com precisão deve ser destacada, independentemente da apresentação clínica do evento isquêmico agudo, medido na primeira consulta médica, especialmente na estratégia fármaco-invasiva.

## References

[1] Montecucco F, Carbone F, Schindler TH (2016). Pathophysiology of ST-segment elevation myocardial infarction: Novel mechanisms and treatments. Eur Heart J.

[2] Armstrong PW, Gershlick AH, Goldstein P, Wilcox R, Danays T, Yves Lambert Y (2013). STREAM Investigative Team Fibrinolysis or Primary PCI in ST-Segment Elevation Myocardial Infarction. N Engl J Med.

[3] Piackova E, Jäger B, Farhan S, Christ G, Schreiber W, Weidinger F, Vienna STEMI Registry Group (2017). Gender differences in short- and long-term mortality in the Vienna STEMI registry. Int J Cardiol.

[4] Kereiakes DJ, Weaver WD, Anderson JL, Feldman T, Gibler B, Aufderheide T (1990). Time delays in the diagnosis and treatment of acute myocardial infarction: a tale of eight cities. Report from the prehospital study group and the Cincinnati Heart Project. Am Heart J.

[5] Ibanez B, Agewall S, Antunes MJ, Bucciarelli-Ducci C, Bueno H, Caforio ALP (2017). 2017 ESC Scientific Document Group, ESC Guidelines for the management of acute myocardial infarction in patients presenting with ST-segment elevation: The Task Force for the management of acute myocardial infarction in patients presenting with ST-segment elevation of the European Society of Cardiology (ESC). Eur Heart J.

[6] Killip T, Kimball JT (1967). Treatment of myocardial infarction in a coronary care unit. a two years experience with 250 patients. Am J Cardiol.

[7] Morrow DA, Elliott M, Antman AC, Charlesworth A, Cairns R, Murphy SA (2000). TIMI Risk Score for ST-Elevation Myocardial Infarction: A Convenient, Bedside, Clinical Score for Risk Assessment at Presentation. Circulation.

[8] Granger CB, Robert J, Goldberg RJ, Dabbous O, Karen S, Pieper KS (2003). Global Registry of Acute Coronary Events Investigators Predictors of hospital mortality in the global registry of acute coronary events. Arch Intern Med.

[9] Chesebro JH, Knatterud G, Roberts R, Borer J, Cohen LS, Dalen J (1985). The Thrombolysis in Myocardial Infarction (TIMI) trial, Phase I findings, TIMI Study Group. N Engl J Med.1985;312(4).

[10] Henriques JPS, Zijlstra F, van Hof AW, de Boer Menko-Jan, Dambrink JHE, Jan-Henk E (2003). Angiographic Assessment of Reperfusion in Acute Myocardial Infarction by Myocardial Blush Grade. Circulation.

[11] Ting HH, Bradley EH, Wang Mr Y, Lichtman JH, Nallamothu BK, Sullivan MD (2008). Factors Associated With Longer Time From Symptom Onset to Hospital Presentation for Patients With ST-Elevation Myocardial Infarction. Arch Intern Med.

[12] Kytö V, Sipilä J, Rautava P (2015). Gender and in-hospital mortality of ST-segment elevation myocardial infarction (from a multihospital nationwide registry study of 31,689 patients). Am J Cardiol.

[13] D’Onofrio G, Safdar B, Lichtman JH, Strait KM, Dreyer RP, Geda M (2015). Sex differences in reperfusion in young patients with ST-segment-elevation myocardial infarction: results from the VIRGO study. Circulation.

[14] Bugiardini R, Yan AT, Yan RT, Fitchett D, Langer A, Manfrini O (2011). Factors influencing underutilization of evidence-based therapies in women. Eur Heart J.

[15] Jneid Hani, Fonarow Gregg C, Cannon Christopher P, Hernandez Adrian F, Palacios Igor F, MareeJneid H Andrew O (2008). Sex differences in medical care and early death after acute myocardial infarction. Circulation.

[16] Lawesson SS, Alfredsson J, Mats F, Swahn E (2012). Time trends in STEMI—improved treatment and outcome but still a gender gap: a prospective observational cohort study from the SWEDEHEART register. BMJ Open.

[17] Stuart E, Sheifer MR, Canos KP, Weinfurt Uk, Umesh K, Farrell A (2000). Sex differences in coronary artery size assessed by intravascular ultrasound. Am Heart J.

[18] Petronio AS, Musumeci G, Limbruno U, Baglini R, Amoroso G, Merelli A (2002). Coronary angioplasty in women: risk factors and sex-related differences in coronary anatomy evaluated with intravascular ultrasonography. Ital Heart J.

[19] Keteepe-Arachi T, Sharma S (2017). Cardiovascular Disease in Women: Understanding Symptoms and Risk Factors. Eur Cardiol.

[20] Mosca L, Benjamin EJ, Berra K, Bezanson J, Dolor RJ, Lloyd-Jones DM (2011). Effectiveness-based guidelines for the prevention of cardiovascular disease in women—2011 update: a guideline from the American Heart Association. Circulation.

[21] de Boer SPM, Roos-Hesselink J, van Leeuwen MAH, Lenzen MJ, van Geuns RJ, Regar E (2014). Excess mortality in women compared to men after PCI in STEMI: an analysis of 11,931 patients during 2000-2009. Int J Cardiol.

[22] Huxley R, Barzi F, Woodward M (2006). Excess risk of fatal coronary heart disease associated with diabetes in men and women: meta-analysis of 37 prospective cohort studies. BMJ.

[23] Tonstad S, Rosvold EO, Furu K, Skurtveit S (2004). Undertreatment and overtreatment with statins: the Oslo Health Study 2000-2001. J Intern Med.

[24] Cull CA, Neil HA, Holman RR (2004). Changing aspirin use in patients with type 2 diabetes in the UKPDS. Diab Med.

[25] Campbell Duncan J, Somaratne Jithendra B, Jenkins Alicia J, Prior David L, Yii Michael, Kenny James F (2011). Differences in Myocardial Structure and Coronary Microvasculature Between Men and Women With Coronary Artery Disease. Hypertension.

[26] Milcent C, Dormont B, Durand-Zaleski I, Gabriel P (2007). Gender differences in hospital mortality and use of percutaneous coronary intervention in acute myocardial infarction: microsimulation analysis of the 1999 nationwide French hospitals database. Circulation.

[27] Cantor WJ, Fitchett D, Borgundvaag B, Ducas J, Heffernan M, Cohen EA (2009). Routine early angioplasty after fibrinolysis for acute myocardial infarction. N Engl J Med.

[28] Mega JL, Morrow DA, Ostör E, Dorobantu M, Qin J, Antman EM, Braunwald E (2007). Outcomes and optimal antithrombotic therapy in women undergoing fibrinolysis for ST-elevation myocardial infarction. Circulation.

[29] Kereiakes DJ, Weaver WD, Anderson JL, Feldman T, Gibler B, Aufderheide T (1990). Time delays in the diagnosis and treatment of acute myocardial infarction: a tale of eight cities. Report from the prehospital study group and the Cincinnati Heart Project. Am Heart J.

[30] Henkel DM, Witt BJ, Gersh BJ, Jacobsen SJ, Weston SA, Meverden RA (2006). Ventricular arrhythmias after acute myocardial infarction: a 20-year community study. Am Heart J.

[31] Rahimi K, Watzlawek S, Thiele H, Secknus MA, Hayerizadeh BF, Niebauer J (2006). Incidence, time course, and predictors of early malignant ventricular arrhythmias after non-ST-segment elevation myocardial infarction in patients with early invasive treatment. Eur Heart J.

[32] Amin AP, Spertus JA, Reid KJ, Lan X, Buchanan DM, Decker C (2010). The prognostic importance of worsening renal function during an acute myocardial infarction on long-term mortality. Am Heart J.

[33] Joachim H Ix, Shlipak MG, Liu HH, Schiller NB, Whooley MA (2003). Association between renal insufficiency and inducible ischemia in patients with coronary artery disease: The Heart and Soul Study. J Am Soc Nephrol.

[34] Turak O, Afsar B, Siriopol D, Yayla C, Oksu F, Cagli K (2016). Severity of coronary artery disease is an independent risk factor for decline in kidney function. Eur J Intern Med.

[35] Vernino S, Brown RD, Sejvar JJR, Petty GW, O’Fallon M (2003). Cause-specific mortality after first cerebral infarction: a population-based study. Stroke.

[36] Dhamoon MS, Tai W, Boden-Albala B, Rundek T, Paik MC, Sacco RL (2007). Risk of myocardial infarction or vascular death after first ischemic stroke: the Northern Manhattan Study. Stroke.

[37] Miettinen H, Lehto S, Salomaa V, Mähönen M, Niemelä M, Haffner SM (1998). Impact of diabetes on mortality after the first myocardial infarction: the FINMONICA Myocardial Infarction Register Study Group. Diabetes Care.

[38] Juutilainen A, Lehto S, Rönnemaa T, Pyörälä K, Laakso M (2005). Type 2 diabetes as a “coronary heart disease equivalent”: an 18-year prospective population-based study in Finnish subjects. Diabetes Care.

