# Supplementary material for: Estratégia Fármaco-Invasiva no Infarto do Miocárdio: Análise Descritiva, Apresentação de Sintomas Isquêmicos e Preditores de Mortalidade
Source: Arq Bras Cardiol. 2022 Nov 1;119(5):691–702. [Article in Portuguese] doi: 10.36660/abc.20211055 (PMC9750212; doi:10.36660/abc.20211055)
Supplement: Translation

# Translation: Pharmaco-invasive Strategy in Myocardial Infarction: Descriptive Analysis, Presentation of Ischemic Symptoms and Mortality Predictors

**Authors:** Henrique Tria Bianco, Rui Povoa, Maria Cristina Izar, Claudia Maria Rodrigues Alves, Adriano Henrique Pereira Barbosa, Maria Teresa Nogueira Bombig, Iran Gonçalves, Bráulio Luna, Ana Caroline Aguirre, Pedro Ivo de Marqui Moraes, Dirceu Almeida, Flávio Tocci Moreira, Fernando Focaccia Povoa, Edson Stefanini, Adriano Mendes Caixeta, Amanda S. Bacchin, Valdir Ambrósio Moisés, Francisco A.H. Fonseca

**Affiliations:** 1 São Paulo SP Brazil Universidade Federal de São Paulo – Cardiologia, São Paulo, SP – Brazil; 2 São Paulo SP Brazil Universidade Federal de São Paulo Escola Paulista de Medicina – Medicina, São Paulo, SP – Brazil

**Keywords:** ST Elevation Myocardial Infarction, Acute Coronay Syndrome, Percutaneous Coronary Intervention/methods, Thrombolytic, Therapy/methods, Angina Pectoris, Hospitalization, Mortality

## Abstract

**Background:**

ST-segment elevation myocardial infarction (STEMI) is defined by symptoms accompanied by typical electrocardiogram changes. However, the characterization of ischemic symptoms is unclear, especially in subgroups such as women and the elderly.

**Objectives:**

To analyze the typification of ischemic symptoms, temporal metrics and observe the occurrence of in-hospital outcomes, in the analysis of predictive scores, in patients with STEMI, in a drug-invasive strategy.

**Methods:**

Study involving 2,290 patients. Types of predefined clinical presentations: typical pain, atypical pain, dyspnea, syncope. We measured the time between the onset of symptoms and demand for care and the interval between arrival at the medical unit and thrombolysis. Odds-ratios (OR; CI-95%) were estimated in a regression model. ROC curves were constructed for mortality predictors. The adopted significance level (alpha) was 5%.

**Results:**

Women had a high prevalence of atypical symptoms; longer time between the onset of symptoms and seeking care; delay between arrival at the emergency room and fibrinolysis. Hospital mortality was 5.6%. Risk prediction by Killip-Kimball classification: AUC: [0.77 (0.73-0.81)] in class ≥II. Subgroups studied [OR (CI-95%)]: women [2.06 (1.42-2.99); p=0.01]; chronic renal failure [3.39 (2.13-5.42); p<0.001]; elderly [2.09 (1.37-3.19) p<0.001]; diabetics [1.55 (1.04-2.29); p=0.02]; obese 1.56 [(1.01-2.40); p=0.04]: previous stroke [2.01 (1.02-3.96); p=0.04] correlated with higher mortality rates.

**Conclusion:**

Despite higher mortality rates in some subgroups, significant disparity persists in women, with delays in symptom recognition and prompt thrombolysis. We highlight the applicability of the Killip-Kimball score in prediction, regardless of the clinical presentation.

## Introduction

Despite advances in reperfusion approaches, acute myocardial infarction continues to be the leading cause of death worldwide. Its diagnosis is considered when typical electrocardiographic (ECG) changes and/or the elevation of markers can be detected, notably troponins, which have received increasing attention as highly specific markers of myocardial injury. ST-segment elevation myocardial infarction (STEMI) is usually caused by acute coronary occlusion, secondary to plaque rupture and thrombosis, requiring early intervention.^1^ Therefore, the management of STEMI must be performed as quickly as possible to prevent further damage to the myocardium and decrease the risk of complications and mortality. Although primary percutaneous coronary intervention (PCI) is considered a “gold standard” treatment, it is not sufficiently available, especially in developing countries. The STREAM study values a reperfusion strategy combining fibrinolytic therapy and immediate transfer to a tertiary center for rescue PCI in non-responders to fibrinolysis, as well as early diagnostic angiography and secondary PCI within 24 h after thrombolysis.^2^ However, some factors can influence the delay in seeking emergency assistance, such as the perception and recognition of acute ischemic symptoms. The interpretation of warning signs is the trigger that leads patients to seek medical attention, due to the severity of this potentially fatal condition. By contrast, pain must be seen as a multidimensional phenomenon involving physiological, sensory, and sociocultural aspects, and can be impacted by expectations within a cultural context.

Risk stratification enables providers to identify the right level of care and services for distinct subgroups of patients. It is the process of assigning a risk status, then using this report to guide what care is provided and to improve overall health outcomes. Nevertheless, during the diagnostic process of myocardial infarction, based on clinical reports and ECG criteria, differences may appear regarding how the symptoms are referred to, particularly in specific subgroups, such as women or older age groups, presenting evidence and relevance of early recanalization, whose benefits become more discreet or even non-existent in late reperfusion.^3,4^ Additionally, there are still controversies and special interests regarding the performance of early mortality predictors in patients undergoing thrombolytic treatment in a pharmaco-invasive approach. Therefore, the present study considers the stratified assessment of ischemic symptoms, fundamentally associated with temporal metrics, including the time between the onset of symptoms and the search for medical care, the patients’ medical needs between arrival at the emergency unit and the recognition of the acute condition, with the prompt installation of the reperfusion protocols. In the scenario of the pharmaco-invasive strategy, possible differences can also be speculated concerning the manner in which the symptoms appeared and pivotal times in some subgroups, considering the impact of late reperfusion on relevant clinical outcomes.

Thus, our study aimed to examine the associations between the clinical presentation of ischemic symptoms and risk factors with cardiovascular outcomes in a cohort of STEMI patients during the hospitalization period, as well as perform an accurate analysis of risk prediction scores.

## Methods

### Study design and ethical statements

Prospective and observational study, with a sample size defined by convenience, involving 2,290 STEMI patients who were consecutively admitted to a university hospital in the city of Sao Paulo, Brazil. All patients were initially submitted to thrombolytic therapy with tenecteplase (TNK) in hospital units or primary care centers and were then referred for coronary angiography. When appropriate, percutaneous coronary interventions were performed within 24 hours post-fibrinolysis, or immediately if rescue therapy was needed. This study complies with the Declaration of Helsinki; the local ethics committee approved the research protocol; and informed consent was obtained from all patients or their legal guardians. The study is registered in ClinicalTrials.gov (NCT02090712).

### Pharmaco-invasive strategy

The pharmaco-invasive strategy is defined by fibrinolysis treatment with a bolus injection of TNK, with a weight-adjusted dose, followed by cardiac catheterization within 24 hours, even in stable patients with successful reperfusion, with the intention to treat the culprit lesion. After the STREAM trial results in June 2013, patients over 75 years of age received a half dose of tenecteplase (1/2 TNK). Upon diagnosis, the patients received acetylsalicylic acid and clopidogrel, as recommended by guidelines.^5^ Rescue angioplasties were recommended by the local medical team due to ineffective thrombolysis in the treatment of an infarct-related artery (IRA). The term *culprit lesion* was used to designate the artery vessel responsible for the symptoms of the STEMI patient. In most cases, only culprit lesions were treated, that is, only IRA lesions were addressed directly through angioplasty and stent delivery. The present study counted on a centralized database, containing a demographic profile, clinical data, ECGs, treatments, time intervals, and in-hospital events. Thus, all relevant outcomes were systematically recorded and mortality rates were analyzed by independent observers.

### Definitions of clinical presentations

The clinical presentation of acute ischemic symptoms was reported by the patients, and trained staff reviewed the data during the in-hospital period.

1. Typical Chest Pain: oppressive chest pain on the left side, which can be irradiated to the left upper limb, of great intensity and prolonged (longer than 20 minutes), which did not improve or had only partial relief with rest or sublingual nitrates. Irradiation to the mandible, right upper limb, back, shoulders, and epigastrium was also considered for this presentation. This group included patients with a concomitant presentation of dyspnea, or syncopal episode.
2. Atypical Pain: pain in the right upper quadrant or epigastric region of the abdomen, dorsal, mandibular region, or another non-thoracic region, referred to as a twinge or burning of variable intensity, with prolonged duration (greater than 20 minutes). Included in this group were patients with a concomitant presentation of dyspnea or syncopal episodes.
3. Dyspnea: this group included those patients who did not report chest pain but who complained of acute fatigue or worsening of this symptom within the last couple of hours. The subjective experience of respiratory distress, comprised of qualitatively different sensations with varying intensity was considered.
4. Syncope: This symptom was considered for those patients who did not report chest pain but did report fainting or a sudden and transient loss of consciousness or any worsening within the last couple of hours.

### Measured pivotal times

1. Time interval between the onset of persistent chest pain, or another representative complaint of ischemic symptoms, and the patient’s arrival at the primary health unit;
2. Time interval between the patient’s arrival at the health unit and thrombolysis;
3. Time interval between thrombolysis and coronary angiography.

### Prediction scores

Risk predictors used during first medical contact:

1. Killip-Kimball Classification (KK);^6^
2. TIMI-Risk;^7^
3. GRACE score^8^

### Angiographic variables

Experienced interventional cardiologists performed angiographic analyses according to TIMI-flow score (epicardial coronary perfusion);^9^ and Myocardial Blush Grade (MBG)^10^ (myocardial tissue-level perfusion), thus obtaining TIMI-flow and MBG before and after the percutaneous intervention when applicable (named as initial and final scores). Complications inherent to the procedure have also been reported. The procedural strategy (thrombus aspiration, balloon dilatation, stent selection, and anticoagulation regimen) was left up to the discretion of the operator.

### Statistical analysis

This study sought to achieve a prospective and consecutive data collection from a large population, in which current standard medical practice is applied in an organized network. Continuous variables were described as mean ± standard deviation (SD) or median and interquartile range [IQR (25th–75th percentiles)], according to the normality of the data. To assess the assumptions of normality, our study used the D’Agostino-Pearson test, developed to evaluate a large sample, and was confirmed through the visual inspection of scatter plots. Categorical variables were described as absolute and relative frequencies, and were examined by Pearson’s Chi-squared test. For comparisons of numerical variables between groups, the unpaired Student’s t-test or the Mann-Whitney U test was used when non-Gaussian distribution was assumed. A simple analysis of variance using the “t” test, or its non-parametric equivalents, were performed to observe the distribution and homoscedasticity of the values. To compare proportions between the groups, the ^χ^² (chi-square) test was used for independent samples. To assume an equality of variance between groups, adjustments were made using Levene’s test. The relative risk was determined by the ratio between carriers of a given variable and non-carriers. To analyze the relationship of some categorical variables and the outcomes, these were transformed into dichotomies. Thus, the proportions test (chi-square) was used to observe the independence between the univariates in order to obtain the odds ratios (ORs) in a correlation model between potentially predictive univariates and the outcomes. In multivariate statistics, the relationships of multiple variables were only verified for those deemed to be significant upon entry (variables with p <0.10) so as to observe their degree of independence. The binary logistic regression model, by means of the maximum likelihood technique, was used, in which the dependent variable was dichotomous and the predictor variables were inserted by the stepwise model, considering the absence of collinearity by the VIF index (variance inflation factor), with the goodness of fit evaluated by the Hosmer-Lemeshow diagram. The predictor variables were analyzed simultaneously in such a way that the effect of each variable was adjusted in order to have an effect on the others. This regression model systematically adds the most significant variable or removes the least significant variable during each step. The standardized Crombach’s ^α^ index was used to calculate the reliability of the TIMI-flow and MBG angiographic scores estimated by medical operators.

Receiver Operating Characteristic (ROC) curves were constructed in order to determine the sensitivity and specificity of the in-hospital outcome prediction scores. The shape of a ROC curve and the area under the curve (AUC) aided in estimating how high the discriminative power of a test was. A perfect diagnostic test has an AUC of 1.0, whereas a non-discriminatory test has an area of 0.5. Other analyses were also applied, considering the likelihood ratios in the prediction of events. Thus, based on the likelihood ratios, the diagnostic odds ratio (DOR), a global measure of diagnostic accuracy, was calculated, that is, the ratio of the odds of positivity in subjects with the outcome to the odds in subjects without the outcome. Our study considered a p-value <0.05 to be statistically significant in two-tailed tests. All analyses were performed using the SPSS, version 20 (IBM-SPSS Statistics, USA)^®^

## Results

### Clinical and epidemiological characteristics

Table 1 shows that patients had a median (IQR) of 58 (50-65) years, and approximately 70% were male. Most patients were hypertensive and smokers, and nearly a third had diabetes. There was a small proportion of patients who presented prior events, such as myocardial infarction, surgical or percutaneous revascularization, and prior stroke. The elderly group were those aged ≥60 years. The risk predictors included data on the patient’s medical history and risk factors, which were reviewed during hospital admission. This study also provides data on hemodynamic variables obtained in the emergency room, such as laboratory data (necrosis and biochemical markers) (Table. 2).

**Table 1:** Baseline characteristics of the studied cohort

Variables	
Epidemiological	Measures
Age; md (IQR) - years	58 (50-65)
Women: md (IQR) - years	60 (52-68)
Men: md (IQR) - years	57 (49-64)
Elderly (≥60 years), n (%)	998 (43,6)
Gender; n (%)	Male: 1607 (70.2)
	Female: 683(29.8)
**Habits – Addictions**	**Measures**
Smoking, n (%)	1472 (64.3)
Alcoholism, n (%)	304 (13.3)
Illicit drugs, n (%)	97 (4.2)
**Clinics**	
Obesity, n (%)	481 (21.0)
*BMI, Kg/m^2^; md (IQR)	26 (23.8-29.3)
Arterial Hypertension, n (%)	1405 (61.4)
Diabetes mellitus, n (%)	661 (28.9)
Dyslipidemia, n (%)	1133 (49.5)
Hypothyroidism, n (%)	145 (6.3)
Chronic kidney disease, n (%)	187 (8.2)
Peripheral arterial disease, n (%)	117 (5.1)
Prior Stroke, n (%)	99 (4.3)
**Chronic coronary syndrome**	**Measures**
Prior myocardial infarction, n (%)	242 (10.6)
Prior coronary angioplasty, n (%)	129 (5.6)
Prior surgical myocardial revascularization, n (%)	45 (2)

Notes: Data on medical history and comorbidities were derived from physician interviews. Demographic information and risk factor profiles were reported by patients, and trained staff reviewed the data during hospitalization. Data are expressed as median (md) and interquartile range (IQR), and categorical variables as frequency (%). For age comparison between genders, the Mann-Whitney non-parametric test was used. Chronic kidney disease (CKD) was estimated by the Modification of Diet in Renal Disease (MDRD) equation and defined when the estimated glomerular filtration rate (eGFR) <60 mL/min/1.73 m^2^; obesity when BMI ≥30; hypertension defined by the use of antihypertensive drugs or systolic blood pressure ≥140 mmHg and/or diastolic blood pressure ≥90 mmHg. Smoking was defined for both ex-smoker and current smokers; dyslipidemia was defined by the use of specific drugs or total cholesterol ≥200 mg/dL, or triglycerides ≥150 mg/dL; diabetes was defined as specific treatment or glycated hemoglobin (HbA1c) ≥6.5%.

* BMI: Body mass index.

**Table 2:** Clinical and hemodynamic characteristics, prediction scores, and pivotal times were obtained during the first medical contact and during the period of hospital stay

Variables obtained during the first medical contact	Measures
*Hemodynamic variables*	
Systolic blood pressure, mmHg; md (IQR)	130 (115-150)
Diastolic blood pressure, mmHg; md (IQR)	80 (70-93)
Heart rate, md (IQR)	76 (66-90)
Killip-Kimball classification, n (%)	Killip-Kimball – I: 1670 (72.0)
	Killip-Kimball – II: 362 (15.8)
	Killip-Kimball – III: 52 (2.3)
	Killip-Kimball – IV: 203 (8.0)
**Clinical presentation** (main symptom), n (%)	Typical Pain: 1939 (88.5)
	Atypical Pain: 166 (7.6)
	Dyspnea: 38 (1.7)
	Syncope: 26 (1.2)
	* Some patients (4%) with more than 1 reported symptom
**Risk Scores**	
TIMI-Risk, (0-14); md (IQR)	3 (2-5)
Grace-escore; md (IQR)	135 (115-160)
**Pivot Times**	
Pain-Health Unit, min; md (IQR)	120 (60-220)
Pain-Needle, min; md (IQR)	222 (140-345)
Door-Needle, min; md (IQR)	71 (42-135)
Lysis-Angiography, hours; md (IQR)	12 (5,67-23)
Variables obtained during the hospital period	Measures
Necrosis biomarkers	
Initial troponin, mg/L; md (IQR)	2655 (538-7967)
Maximum troponin, mg/L; md (IQR)	4718 (1481-9842)
**Laboratory variables**	
Hemoglobin / Hematocrit, g/dL / %; m ± SD	14.37 ± 2,09 / 42.92 ± 12.56
Blood glucose / Glycated hemoglobin, mg/dL / %; md (IQR)	122 (102-160) / 6 (5.6-6.8)
Total Cholesterol, mg/dL; md (IQR)	191 (157-225)
HDL-C, mg/dL; md (IQR)	37 (25-46)
LDL-C, mg/dL; md (IQR)	110 (60-142)
Triglycerides, mg/dL; md (IQR)	118 (77-175)
*AST, u/L; md (IQR)	144 (63-280)
†ALT, u/L; md (IQR)	43 (27-72)
Creatinine, mg/dL; md (IQR)	0,9 (0.74-1.10)
Estimated Glomerular Filtration Rate, (MDRD); md (IQR)	85 (64-107)

Notes: Data are expressed as median (md) and interquartile range (IQR), or mean and standard deviation (m ± sd), and categorical variables are presented as frequency (%). Time metrics are expressed in minutes (min). Estimated Glomerular Filtration Rate by Modification of Diet in Renal Disease (MDRD)

* AST: aspartate aminotransferase

† ALT: alanine aminotransferase.

### Prediction scores, clinical presentation and pivotal times

In the analysis of ischemic symptoms, most patients reported chest pain defined as typical, which may or may not be associated with dyspnea or syncope. Angina prior to the event was present in 28%, which was more prevalent in patients with prior myocardial infarction. Women showed a high frequency of atypical symptoms, such as dyspnea and syncope, as shown in Table 3.

**Table 3:** Variables associated with the type of clinical presentation in a univariate model and after multivariate adjustments in multinomial logistic regression

Model without adjustments	Typical Pain	Atypical Pain	Dyspnea	Syncope
Variables	**Odds-ratio** (95% CI), p-value	**Odds-ratio** (95% CI), p-value	**Odds-ratio** (95% CI), p-value	**Odds-ratio** (95% CI), p-value
	n = 1939	n = 166	n = 38	n = 26
Male	0.95 (0.71-1.27), p=0.74	1.05 (0.79-1.40), p=0.73	**0.51** (0.27-0.96), p=0.026	**0.39** (0.18-0.83), p=0.018
Obesity	1.07 (0.78-1.50), p=0.65	0.92 (0.67-1.28), p=0.65	**0.30** (0.09-0.98), p=0.02	0.29 (0.07-1.26), p=0.09
Alcoholism	0.80 (0.56-1.15), p=0.24	1.24 (0.86-1.78), p=0.24	1.15 (0.48-2.77), p=0.44	1.49 (0.56-3.97), p=0.39
Hypertension	0.96 (0.73-1.26), p=0.78	1.03 (0.79-1.35), p=0.79	1.31 (0.67-2.56), p=0.26	0.67 (0.31-1.44), p =0.32
Dyslipidemia	**1.39** (1.07-1.81), p=0.013	**0.71** (0.55-0.93), p=0.013	1.25 (0.66-2.35), p=0.52	**0.35** (0.15-0.84), p =0.019
Prior myocardial infarction	1.05 (0.68-1.61), p=0.81	0.95 (0.62-1.46), p=0.82	**3.31** (1.63-6.72), p=0.002	0.67 (0.16-2.86), p =0.59
Prior Stroke	0.75 (0.42-1.34), p=0.34	1.32 (0.74-2.37), p=0.34	1.82 (0.55-6.00), p=0.24	0.85 (0.11-6.32), p =0.87
Peripheral artery disease	0.78 (0.45-1.35), p=0.30	1.27 (0.74-2.20), p=0.38	1.52 (0.46-5.00), p=0.45	0.71 (0.09-5.29), p =0.74
Chronic kidney disease	**0.53** (0.35-0.79), p=0.002	**1.88** (1.26-2.80), p=0.002	**4.47** (2.19-9.10), p<0.001	1.97 (0.67-5.77), p =0.27
Smoking	1.17 (0.90-1.53), p=0.23	0.85 (0.65-1.11), p=0.23	1.15 (0.59-2.25), p=0.74	0.59 (0.27-1.27), p =0.22
Diabetes	0.95 (0.72-1.27), p=0.76	1.04 (0.78-1.39), p=0.75	**2.26** (1.21-4.24), p=0.013	0.70 (0.28-1.74), p =0.52
Elderly	0.87 (0.67-1.13), p=0.32	1.14 (0.88-1.48), p=0.32	1.77 (0.94-3.33), p=0.07	0.76 (0.34-1.66) p =0.56
**Adjusted model**	**Typical Pain**	**Atypical Pain**	**Dyspnea**	**Syncope**
Variable	**Odds-ratio** (95% CI), p-value	**Odds-ratio** (95% CI), p-value	**Odds-ratio** (95% CI), p-value	**Odds-ratio** (95% CI), p-value
Males	NA	NA	0.51 (0.26-0.97), p=0.04	0.32 (0.15-0.70), p=0.005
Obesity	NA	NA	0.29 (0.08-0.95), p =0.04	NA
Dyslipidemia	**1.44** (1.10-1.87), p=0.007	**0.69** (0.53-0.90), p =0.007	NA	**0.36** (0.15-0.87), p=0.02
Prior myocardial infarction	NA	NA	2.68 (1.28-5.58), p=0.008	NA
Chronic kidney disease	**0.50** (0.34-0.75), p=0.001	**1.97** (1.32-2.94), p=0.001	**3.33** (1.59-6.98), p=0.001	NA
Diabetes Mellitus	NA	NA	**1.93** (1.01-3.71), p=0.04	NA

Notes: Data are expressed for (OR; 95% CI. p-value). In the multivariate analysis the predictor variables were analyzed simultaneously, so that the effect of each variable was adjusted for the effect of the others. Bold indicates statistical significance. Chronic kidney disease (CKD) was estimated by Modification of Diet in Renal Disease (MDRD); elderly: age ≥60 years. Bold indicates statistical significance. NA: not applicable.

Women presented a delay between the patient’s arrival at the emergency unit and the beginning of treatment: [women (2 h:17 min.) *vs* men (1 h:58 min.), p=0.021]. The stratified time (≥240 min.) to receive treatment was favorable to the males: [OR 0.73; 95% CI (0.55-0.98), p=0.03]. Another relevant finding was the longer time interval between the onset of symptoms and the search for medical care among women: [women: (3 h:14 min.) vs men (2 h:48 min.), p=0.008]. During this period, diabetic women presented a longer time interval between the onset of symptoms and thrombolysis, including the arrival at the emergency unit at the beginning of treatment (pain-thrombolysis time), especially when compared to non-diabetic men (Figure 1).

**Figure 1:** Time metrics related to gender and presence or absence of diabetes. Note. Pain-needle time expressed as mean (minutes). * Significant p-value when comparing non-diabetic men versus women with diabetes.

Most patients were in a low functional class, according to the KK score: [I (73%), II (16.3%), III (2.2%), and IV (8.6%)]; low-risk profile in the TIMI-Risk predictions score: [3, IQR (2-5)]; and GRACE: [136, IQR (117-161)]. Approximately 24% of the patients were referred for rescue angioplasty, as judged by the local medical team, for not achieving criteria for successful initial reperfusion therapy.

### Angiographic findings (culprit artery)

1. left anterior descending artery (LAD): 46.3%; 2) right coronary artery (RCA): 32.1%; 3) left circumflex artery (LCX): 6.1%; 4) left main coronary artery (LMCA): 0.4%; 5) posterior descending artery (PDA): 0.3%; 6) posterior ventricular branch (PV): 0.9%; 7) left marginal branch: 1.0%; 8) diagonal branch: 0.7%; 9) unidentified artery: 12.1%. The subgroup analysis found no significant associations between the culprit artery and prior risk factors, nor with the initial clinical presentation of ischemic symptoms.

The TIMI-flow and MBG (initial and final) scores were recorded, ranked from 0 to 3: [TIMI-initial flow: (3)] and [MBG-initial: (3)] in approximately 60% and 42%, respectively. When these post-percutaneous procedure scores were analyzed, the [TIMI-final flow: (3)] and the [MBG-final: (3)] rates were: 78% and 58%, respectively, with a high level of reliability: Crombach’s **α** =0.88.

### Outcomes associated with complications in the hemodynamics laboratory and by the index event

The average length of hospital stay was 2.0 ± 1.3 days, from admission to tertiary hospital up to discharge or transference to a counter-referenced hospital to continue the treatment. A longer hospital stay was observed in the elderly group: [1.9 days (non-elderly) *vs.* 2.3 days (elderly), p=0.004] and notably in the group of patients who required replacement blood products due to bleeding: [1.9 days (without bleeding) vs. 3.4 days (major bleeding), p=0.004].

The present study recorded the frequency of complications during the angiographic procedure, as well as those observed during the hospitalization period. These complications included important events, such as coronary dissection or rupture, among others, such as clinical complications, arrhythmias, and bleeding at the puncture site. Although infrequent, major bleeding was independently associated with in-hospital mortality. Higher bleeding rates were observed in the elderly group: [OR: 1.86; 95% CI (1.04-3.16), p=0.023].

### Predictors and variables associated with mortality rates

The in-hospital mortality rate was 5.6%, with 128 deaths; of these, 23 (17.9%) occurred in the hemodynamics laboratory, with electrical instability or mechanical complications being the most prevalent and with a higher incidence occurring in the rescue group (11.5% *vs.* 2.4%). Regarding epidemiological characteristics and risk factors, a similar distribution of the analyzed variables was observed among surviving patients, when compared with those who died during the hospital period, except for the GRACE score, which showed higher values in patients who died: [134 (115-157) *vs.* 202 (155-233), p<0.001]. The GRACE score (median score 136) showed good sensitivity but low specificity [sensitivity: 0.86%; specificity: 0.53%]. The TIMI-Risk Prediction Score (median score 3) presented the following: sensitivity: 0.87%; specificity: 0.57% (Table 4).

**Table 4:** Clinical and epidemiological variables between the “survivor” and the “death” groups

	Survivor group	Death group	p-value
**Variables**	2162 (94.4%)	128 (5.6%)	
**Epidemiological**			
Age, years:	58 (50-66)	56 (48-65)	**0.047**
Male gender:	Homens: 71.1%	Homens: 53.2%	**0.02**
**Risk Scores**			
Killip-Kimball, (%):	Killip-Kimball - I: 75.6%	Killip-Kimball- I: 20.6%	0.09
	Killip-Kimball - II: 16.4%	Killip-Kimball- II: 7.9%	0.42
	Killip-Kimball - III: 2.2%	Killip-Kimball- III: 4.8%	0.96
	Killip-Kimball - IV: 5.7%	Killip-Kimball- IV: 66.5%	0.08
TIMI-Risk:	3 (2-5)	6 (5-8.2)	0.26
GRACE-score:	134 (115-157)	202 (155-233)	**p <0.001**
**ECG-variables**			
ECG wall	anterior wall	anterior wall	anterior wall
	inferior wall	inferior wall	inferior wall
	lateral wall	lateral wall	lateral wall
**Hemodynamic Variables**			
Infarct-related artery:	ADA: 45.5%	ADA: 38.9%	0.84
	ACD: 32.5%	ACD: 36.5%	
	ACX: 6%	ACX: 10.3%	
	Others: 22.4%	Others: 14.3%	
†Left ventricular ejection fraction (LVEF):	50 (40-59)	49 (40-60)	0.17
Initial TIMI-flow:	TIMI-0: 19.6%	TIMI-0: 36.8%	0.97
	TIMI-1: 3.3%	TIMI-1: 11.3%	
	TIMI-2: 15.4%	TIMI-2: 17%	
	TIMI-3: 61.7%	TIMI-3: 34.9%	
Final TIMI-flow:	TIMI-0: 4.6%	TIMI-0: 19.8%	0.10
	TIMI-1: 1.2%	TIMI-1: 4.7%	
	TIMI-2: 14.1%	TIMI-2: 24.5%	
	TIMI-3: 80.1%	TIMI-3: 50.9%	
Initial myocardial Blush-grade:	Blush-0: 40.8%	Blush-0: 71.7%	0.77
	Blush-1: 3.8%	Blush-1: 2.8%	
	Blush-2: 2.6%	Blush-2: 1.9%	
	Blush-3: 52.8%	Blush-3: 23.6%	
Final myocardial Blush-grade:	Blush-0: 24.7%	Blush-0: 61%	0.39
	Blush-1: 8.8%	Blush-1: 9.5%	
	Blush-2: 6.1%	Blush-2: 5.7%	
	Blush-3: 60.4%	Blush-3: 23.8%	
**Laboratory Variables**			
Initial troponin (at baseline):	2704 (618-7889)	3413 (280-11506)	0.83
Maximum troponin:	4820 (1661-9796)	7925 (1145-1774)	0.80
*eGFR, (MDRD):	86 (67-107)	91 (66-111)	0.62
Hemoglobin:	14.5 (13.3-15.7)	13.7 (12.5-15.1)	0.05
Hematocrit:	42.9 (39.6-46.2)	41 (37.9-45.5)	0.14
**Pivot Times**			
Pain-Needle Time, (min):	220 (140-345)	245 (150-516)	0.08
Door to Needle Time, (min):	75 (45-135)	78.5 (45-163.7)	0.12
‡Lyse-CATHETERISM TIME, (hours):	11 (5-22)	11 (5-21.7)	0.43

Notes: Data are expressed as median (md) and interquartile range (IQR), and categorical variables are expressed as frequency (%). The χ² (chi-squared) test was used for the independent samples.

* eGFR: estimated glomerular filtration rate by Modification of Diet in Renal Disease (MDRD).

† LVEF: Left Ventricular Ejection Fraction.

‡ Lyse-CATHE (fibrinolysis-catheterization); ECG: electrocardiographic. Bold indicates statistical significance.

The KK functional classification showed good performance in predicting in-hospital mortality: AUC: [0.77 95% CI (0.73-0.81), p<0.001] in the group with a score ≥ II, showing better accuracy than the reperfusion angiographic scores: TIMI-Flow (3) and MBG (3), AUC: [0.69; 95% CI (0.64-0.75), p<0.001], as well as a better performance when we stratified the patients, according to left ventricular ejection fraction, AUC: [0.52; 95% CI (0.47-0.58), p=0.34].

Our study was careful to evaluate the performance of the prediction scores: TIMI-Risk, AUC: [0.79; 95% CI (0.75-0.84)], p<0.001; GRACE, AUC: [0.82; 95% CI (0.78-0.86), p<0.001]; Killip-Kimball AUC: [0.82; 95% CI (0.78-0.87), p<0.001], (Figure 2). For the Killip-Kimball categories, the following scores were obtained: positive likelihood ratio: 3.76; negative likelihood ratio: 0.33; and DOR (Diagnostic Odds Ratio): 11.39 for in-hospital mortality prediction rates, defined as the probability of patients in the functional class ≥ II (II, III, IV) who died, relative to the probability of patients in the functional group ≥ II who survived.

**Figure 2:** Prediction scores for in-hospital mortality. Note. The C-Statistics, ROC and AUC. TIMI-Risk, AUC: [0.79; 95% CI (0.75-0.84), p<0.001]; GRACE-score, AUC: [0.82; 95% CI (0.78-0.86), p<0.001]; Killip-Kimball, AUC: [0.82; 95% CI (0.78-0.87), p<0.001].

In a logistic regression model with covariance analyses, obesity, women, patients with diabetes mellitus, chronic renal failure, previous stroke, and the elderly were associated with the highest rates of fatal events (Figure 3). The model fit presented a good predictive performance.

**Figure 3:** In-hospital mortality predictors. Note. Prediction variables in a binary logistic regression model, with OR-95% CI; and p-value.

## Discussion

Even with the existence of many effective therapies, qualitative information is still lacking for stratification, notably in the pharmaco-invasive strategy, where the initial health assessment seems closely related to the prognosis. There are many approaches to risk stratification. Some are very complex and costly, but simple procedures can also be effective. In this light, the present study sought to provide descriptive epidemiological data on the different forms of acute ischemic symptoms, as well as show the applicability of some prediction scores in patients who received medical care mainly in basic health units and were subsequently referred to a tertiary university hospital for angiographic study and invasive procedures.

Not every acute coronary syndrome shows classic signs or symptoms, such as the typical precordial or retrosternal pain, in turn delaying the diagnosis and therapeutic approaches, and impacting the prognosis. Thus, there is great interest in the major determinants of mortality and short-term complications after an acute myocardial infarction.^11^ In recent years, major advances in diagnosis and treatment have contributed to the decline in mortality due to coronary heart disease. In fact, efforts have been made for more active primary prevention, through a better control of risk factors, lifestyles, and pharmacological resources. Nevertheless, challenges regarding the prevention and treatment of cardiovascular diseases need to be widely available and applied without distinction.

### Clinical presentation and events in subgroups

There is evidence of differences among genders in the application of technological advances.^12–15^ In our analysis, symptoms referred to as dyspnea or syncopal episodes were significantly more prevalent in women, and were particularly relevant for this group, presenting higher mortality rates. Several multifactorial mechanisms have been proposed for the greater cardiac mortality of women. The explanations often include a smaller coronary diameter in females, lower collateral flow, predisposition to plaque erosion and distal embolization, and other phenotypic characteristics of atherosclerotic plaques.^16–18^ Women generally present events approximately a decade later than men, notably in post-menopause, possibly due to an estrogenic decrease and a loss of protective effects, with a consequent worsening of risk factors, weight gain, and insulin resistance.^19–21^ In our cohort, the delay in recognizing symptoms, possibly due to their atypical appearance, had an impact on temporal metrics for thrombolysis, such as those recorded in the group of women and diabetics.

Diabetes is a potential risk factor in women. A meta-analysis to estimate the relative risk of fatal coronary heart disease associated with diabetes, involving approximately 450,000 patients, revealed a 50% higher relative risk in women.^22^ The higher coronary risk associated with diabetes in women may reflect a treatment bias favorable to men. Studies show that men with diabetes or established cardiovascular disease are more likely to receive antiplatelet drugs, statins, or antihypertensive drugs than women.^23,24^ Additionally, there are reports of poor adherence to guideline recommendations in women, such as a longer door-to-balloon time.^25–28^ It was also observed that males received fibrinolytic therapy earlier, possibly due to the clearer presentation of ischemic symptoms.

The clinical appearance, defined above as dyspnea, was more prevalent in patients with previous infarctions, possibly due to additional myocardial impairment. The report of dyspnea was also noted in patients with diabetes. This may have had a significant impact on the mortality rate due to the absence of typical symptoms or warning signs.

### Risk scores

Although there is no ideal stratification model, it should contain the following characteristics: easy implementation, objectivity, accuracy, and widespread use. Killip-Kimball, a functional classification method applied during first medical contact, was an important predictor for fatal outcomes during the hospital period, with a good negative predictive value. The index of the severity of cardiac insufficiency in patients with acute myocardial infarction was proposed by Thomas Killip and John Kimball in an attempt to measure the risk of in-hospital events and the potential benefit of the specific management of medical care provided in coronary care units. Our analysis highlights the clinical use of a physical examination as a simple tool without sophisticated technological requirements in order to identify signs of heart failure upon hospital admission, which played a relevant prognostic role in mortality rates for the hospital period, since the proportions of deaths and the distributions of survival data were significantly different within Killip-Kimball class > I.

### Hospital mortality rates

The mortality rate, including events during the angiographic procedure and those related to the index event, was associated with mechanical complications and severe and irreversible electrical disturbances. It has been reported that delays in recanalization time are associated with a greater impairment of ventricular function, microcirculation disturbances, and higher mortality rates.^29^ It is interesting to note that our study found no association of mortality rates among patients in the group with lower left ventricular ejection fraction (LVEF) nor with the angiographic scores. In fact, totally occluded culprit lesion (TIMI-flow-0) proved to be unassociated with higher in-hospital mortality rates after STEMI treated with TNK, when compared to TIMI-flow ≥1. Moreover, malignant ventricular arrhythmias can appear earlier in ischemic processes and continue to be an expected cause of death in myocardial infarctions.^30,31^ In our cohort, in-hospital mortality rates were higher among patients with sustained malignant ventricular arrhythmias than among patients without sustained ventricular arrhythmias, defined as ventricular tachycardia or fibrillation. However, due to the observational characteristic of our study, several difficulties were encountered in characterizing the ventricular arrhythmias, especially in the post-angioplasty period, as the occurrence of these events was difficult to predict. Thus, it can be speculated that severe electrical disturbances could be a strong marker of in-hospital outcomes, despite the success of percutaneous coronary intervention and their respective angiographic scores, and they may not be correlated with LVEF as well. Presumably, it seems to be a better marker when applied to measure outcomes in the medium and long terms.

Some subgroups of special interest were examined in their associations with mortality rates, such as women, obese patients, diabetics, and elderly individuals. In this sense, after myocardial infarction, women appear to be at a higher risk of reinfarction and death, which can be partially explained by the more advanced age, as observed in our study.

By contrast, chronic kidney disease (CKD) represents an independent risk factor for the development of ischemic heart disease, increasing mortality with the advancement of renal impairment. Previous kidney damage to or as a consequence of infarction is associated with worse outcomes.^32,33^ It is likely that there is a reciprocal relationship between the coronary atherosclerotic process and the renal function, and that the presence of coronary disease would be associated with a worsening of the renal function. CKD and cardiovascular disease are closely related, and the presence of one condition synergistically affects the prognosis of the other.^34^ Our data express the highest mortality rates for these patients.

Patients who had suffered a prior stroke also showed higher rates of fatal events, highlighting the need for prevention and adequate care for this subgroup.^35,36^ The same concept may be applicable for elderly individuals, possibly due to the greater biological frailty in this group. The mechanisms for this relation seem to be multiple, involving anatomical, biochemical, and immunological characteristics, or even longer exposure to classic risk factors. Another finding in our cohort was the higher mortality rates in patients with diabetes mellitus, which is in agreement with previous studies.^37–39^ Diabetes mellitus is often associated with multiple mechanisms for cardiovascular disease, such as obesity, hypertension, renal failure, subclinical inflammation, endothelial dysfunction, and microvascular impairment. Nonetheless, no significant differences were found in either the TIMI-flow or the MBG reperfusion angiographic scores when comparing the groups of patients with and without diabetes.

### Strengths and limitations

The present study has limitations. First, it was an observational study, with correction of known or measured confounding factors. Thus, we cannot conclude that the observed associations are causal. Furthermore, our records included only patients who underwent pharmaco-invasive intervention, disregarding those who were referred for primary percutaneous treatment or those who had formal contraindications for fibrinolysis. Given that the study was performed at a single university center, these standards of practice and results should be interpreted with caution. Another limitation of our study was the analysis of outcomes for the in-hospital period only. In an important sense, our internal validation indicated that the model fit was good and the diagnostic prediction models performed well in independently predicting the prognosis.

## Conclusions

Our data revealed higher in-hospital mortality rates in women; in patients with diabetes mellitus, obesity, CKD, and prior stokes; as well as in the elderly. The significant gender-related disparity persists in women, with delays in the recognition of symptoms of ischemia, and the immediate initiation of fibrinolytic therapy, thus favoring worse clinical results. The applicability of the Killip-Kimball score in accurately predicting fatal events should be highlighted, regardless of the clinical presentation of the acute ischemic event, measured upon the first medical contact, especially in the pharmaco-invasive strategy.
